# Prospective multicenter study of heart rate variability with ANI monitor as predictor of mortality in critically ill patients with COVID-19

**DOI:** 10.1038/s41598-022-25537-z

**Published:** 2022-12-16

**Authors:** Cristian Aragón-Benedí, Andres Fabricio Caballero-Lozada, Angel Augusto Perez-Calatayud, Angela Maria Marulanda-Yanten, Pablo Oliver-Fornies, Emmanuel Boselli, Julien De Jonckheere, Sergio D. Bergese, ANI-COVID-19 Research Group, Javier Martinez-Ubieto, Ana Pascual-Bellosta, Sonia Ortega-Lucea, Juan Pablo Quintero Fernandez, Miguel Ángel Martínez Camacho, Leidy Gaviria-Villarreal, Jorge Mejia Mantilla, Irene Lopez-Arribas, Alejandro Centeno-Perez, Margarita Merino-Ruiz, Raquel Fernandez-Garcia, Mario Fajardo-Perez, Stanislas Ledochowski

**Affiliations:** 1 Department of Anesthesia, Resuscitation and Pain Therapy, Miguel Servet University Hospital, Zaragoza, Spain; 2 Departamento de Anestesiología, Hospital Universitario del Valle, Universidad del Valle, Cali, Colombia; 3 Áreas Críticas Hospital General de México Dr Eduardo Liceaga, Mexico, Mexico; 4 Fundación Valle del Lili, Cali, Colombia; 5 Department of Anesthesia, Resuscitation and Pain Therapy, Mostoles General University Hospital, Madrid, Spain; 6 Department of Anesthesiology, Centre Hospitalier Pierre Oudot, Bourgoin-Jallieu, France; 7 CIC-IT 1403, Lille University Hospital, Lille, France; 8 Stony Brook University Hospital, New York, USA

**Keywords:** Neuroscience, Medical research, Biomarkers

## Abstract

The purpose of this study is to demonstrate that the most critically ill patients with COVID-19 have greater autonomic nervous system dysregulation and assessing the heart rate variability, allows us to predict severity and 30-day mortality. This was a multicentre, prospective, cohort study. Patients were divided into two groups depending on the 30-day mortality. The heart rate variability and more specifically the relative parasympathetic activity (ANIm), and the SDNN (Energy), were measured. To predict severity and mortality multivariate analyses of ANIm, Energy, SOFA score, and RASS scales were conducted. 112 patients were collected, the survival group (n = 55) and the deceased group (n = 57). The ANIm value was higher (p = 0.013) and the Energy was lower in the deceased group (p = 0.001); Higher Energy was correlated with higher survival days (p = 0.009), and a limit value of 0.31 s predicted mortalities with a sensitivity of 71.9% and a specificity of 74.5%. Autonomic nervous system and heart rate variability monitoring in critically ill patients with COVID-19 allows for predicting survival days and 30-day mortality through the Energy value. Those patients with greater severity and mortality showed higher sympathetic depletion with a predominance of relative parasympathetic activity.

## Introduction

At the end of 2019, the world faced a new virus, severe acute respiratory syndrome coronavirus 2 (SARS-CoV-2), which led to the coronavirus disease 2019 (COVID-19)^1^. COVID-19 mainly involves the respiratory system^2^, although in more severe stages it affects the whole system through a significant humoral and cellular inflammatory response^3^. It is characterized by a retrograde axonal transport and direct viral invasion of the neural parenchyma, which, along with this systemic inflammatory response, could be the mechanism for different neurological and autonomic manifestations in COVID-19 patients^4^.

Heart rate variability (HRV) analyzes the oscillation in the interval between consecutive heartbeats and is a biomarker of cardiac autonomic control widely used to assess the autonomic nervous system (ANS) activity. Dysfunction of the ANS is a maladaptive response, and therefore, the measurement of HRV, as a biomarker of sympathovagal balance, is proposed as a noninvasive model for risk stratification^5,6^.

Over the last year, several articles have studied the autonomic dysregulation in patients infected with SARS-CoV-2 and have analyzed how spectral analysis of HRV can be used to assess autonomic function in patients at different stages of COVID-19 disease progression^7–9^. The impact of SARS-CoV-2 on different neurological mechanisms, however, remains unknown^10^.

The Analgesia Nociception Index (ANI) monitor (MDoloris Medical Systems, Loos, France) is a medical device allowing the assessment of the autonomic effect of nociception and analgesia using HRV analysis. This device is used worldwide to monitor nociception/antinociception imbalance during surgery or in intensive care units (ICU). It allows displaying two indexes; the ANI, which is related to the parasympathetic activity, and the Energy, which represent the global ANS activity.

The main objective of this study is to demonstrate that the most critically ill patients with COVID-19 have greater ANS dysregulation while assessing ANI and Energy ability to predict 30-day mortality, severity, and days of survival in critically ill patients under mechanical ventilation with COVID-19. In addition, other objectives were to correlate ANI and Energy with different proinflammatory cytokines, such as IL-6, C-reactive protein (CRP), and procalcitonin, and to correlate this biomarker with the Sequential Organ Failure Assessment (SOFA scale) for multi-organ failure and Richmond Agitation-Sedation scale (RASS) for analgosedation used in the critically ill patient.

## Material and methods

### Study design and setting

This was a multicenter, prospective, cohort study involving five hospitals in four different countries (Hospital General de Mexico [Mexico], Hospital Universitario del Valle [Colombia], Fundación Valle del Lili [Colombia], Centre Hospitalier Pierre Oudot [France], and Mostoles General University Hospital [Spain]), conducted between September 2020 and September 2021. It was approved by the Ethical and Research Committee of Mostoles General University Hospital (Madrid, Spain), registered No. 2020/035. A pilot study assessing the feasibility of adherence protocols and the convenience of the study in COVID-19 patients admitted to the Surgical Intensive Care Unit, was previously published^9^.

This study was performed in line with the principles of the Declaration of Helsinki and written informed consent was obtained from all the institutions and all the subjects by the legal designees. This report follows the Strengthening the Reporting of Observational Studies in Epidemiology reporting guideline (STROBE).

### Inclusion/exclusion criteria

We included patients aged ≥ 18 years with severe COVID-19 disease (diagnosed by a positive polymerase chain reaction [PCR] test for SARS-CoV-2) admitted to the Surgical Intensive Care Unit on mechanical ventilation, through orotracheal intubation or tracheostomy. The exclusion criteria were patients with pacemakers or sinus rhythm arrhythmia.

### Primary and secondary outcomes

The primary outcome was the analysis of the difference in HRV between groups, and more specifically the analysis of the relative parasympathetic activity, i.e., Analgesia Nociception Index (ANI), and of the standard deviation of all normal R–R intervals (SDNN), i.e., Energy, provided by the ANI monitor. The secondary outcome was to attempt to predict severity and mortality by the univariate and multivariate analysis of the ROC curves of the analysis of ANI, Energy, and other prognostic variables (age, SOFA scale, RASS scale).

### Heart rate variability and analgesia nociception index computation

HRV refers to the variation between one heartbeat and the next, i.e., R-R interval on an ECG, a process that is influenced by different components of the ANS, including breathing and other physiological factors^11,12^.

Analysis of the different frequency and time domain indices of HRV is a non-invasive method that evaluates the activity of the ANS^6,11,12^. In the time domain, the SDNN expresses the overall HRV and reflects the overall activity of the ANS, calculated in seconds (s). In the frequency domain, the high-frequency (HF) component, between 0.15 and 0.4 Hz, is mediated by the parasympathetic nervous system which is mainly influenced by the respiratory sinus arrhythmia (RSA); the low-frequency (LF) component, between 0.04 and 0.15 Hz, is mainly influenced by the sympathetic nervous system and baroreflex mechanisms, although the influence of parasympathetic activity is also partially present; and, lastly, the very-low-frequency (VLF) component, between 0.003 and 0.04 Hz, is influenced by thermoregulation and different hormonal factors^13^. The HF variations of heart rate can also be expressed in a normalized unit (HFnu) and are usually defined as the ratio between the absolute value of the HF and the sum of HF and LF, i.e. HFnu = HF/(HF + LF). It, therefore, expresses the amount of parasympathetic variation over to the whole variability excluding VLF^6,14–16^.

The parameters of HRV were recorded using the ANI monitor (MDoloris Medical Systems, Loos, France). The ANI monitor displays the Energy value which is equivalent to the SDNN index. Indeed, it exists a linear mathematical relation between Energy and SDNN as shown in the following formulas:

$${\text{Energy = }}\sqrt {\sum\limits_{{{\text{i}} = 1}}^{{\text{N}}} {\left( {{\text{RR}}_{{\text{i}}} - {\text{RR}}_{{{\text{mean}}}} } \right)}^{2} } \;\;{\text{SDNN = }}\sqrt {\frac{{\sum\limits_{{{\text{i}} = 1}}^{{\text{N}}} {\left( {{\text{RR}}_{{\text{i}}} - {\text{RR}}_{{{\text{mean}}}} } \right)}^{2} }}{N}} \;\;Energy = SDNN*\sqrt N$$

ANI is an index related to the relative parasympathetic activity which analyses the magnitude of the normalized HF oscillations. It is computed by a graphical method calculating the area under the curve (AUC) of the oscillations produced by the RSA, i.e., HF oscillations, as it has been described by Logier et al.^15^ and Jeanne et al.^16^.

**Figure 1:**
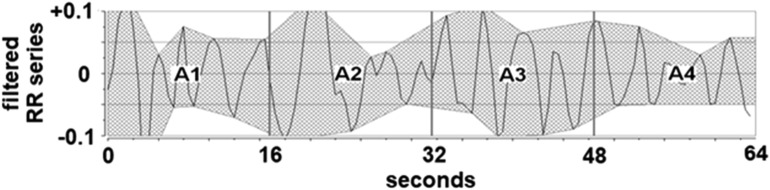
Centered, normalized and filtered RR series. A1, A2, A3 and A4 are the areas measuring the respiratory influence in the RR series.

For Energy and ANI computation, the RR intervals are isolated in a 64 s moving window. The mean RR value and the Energy are computed in this window. RR series is then centered by subtracting the mean value from each RR interval and normalized by dividing each RR interval by the Energy. The mean-centered RR series is then filtered between 0.15 and 0.4 Hz in order to obtain a signal representative of the HF oscillations. The envelopes between local maximum and minimum are then plotted (Fig. 1) and the area between the upper and lower envelopes is computed.

The area is then divided into 4 sub-areas A1, A2, A3, and A4 and AUCmin is defined as the lower sub-area.

AUCmin = min (A1, A2, A3, A4). ANI is then defined as ANI = 100 × (a × AUCmin + b)/12.8 where a = 5.1 and b = 1.2 were determined on a 200 patients dataset to get a value between 0 and 100. ANI is then averaged over 56 s in order to obtain the instantaneous ANI (ANIi) and over 176 s in order to obtain the mean ANI (ANIm).

Thus, the ANI monitor provides a number from 0 to 100, which represents the normalized HF oscillations magnitude^14–16^. This index is, therefore, strongly correlated to HFnu.

The ANI monitor only provides Energy and ANI parameters and doesn’t allow the computation of VLF, LF, HL, and HFnu. In this study, HRV analysis will therefore be limited to ANI and Energy.

### Measurements and data handling

HRV was recorded using the ANI monitor. For this, the specific ANI monitor electrodes for ECG were placed on the patient’s chest or back, depending on whether the patients were in a supine or prone position. ANIm and mean Energy values were collected for 300 s (5 min), from a single measurement in the morning before daily washing. Any changes in drugs potentially affecting HRV and invasive procedures were avoided too.

Patient demographic data included age, weight, height, BMI, and comorbidities (heart disease, chronic beta blockers treatment, lung disease, immunosuppression, diabetes mellitus, hepatic disease, renal disease, obesity, and neurological disease). Drugs used for analgosedation (midazolam, propofol, dexmedetomidine, ketamine, fentanyl, remifentanil, morphine, and neuromuscular blocking drugs), corticoids, anticoagulants, antibiotics, and the need for vasoactive drugs (norepinephrine and/or dobutamine) were also recorded.

To assess the degree of sedation, the RASS scale^12,17,18^ was used, and electroencephalogram monitoring with bispectral index (BIS), if it was available at the institution^17,18^. To estimate the severity, the SOFA score was used – the gold-standard score for organ failure in critically ill patients^19^. A blood test for CRP, procalcitonin, and IL-6, was performed on the morning of the same day of the HRV measure and the values were obtained from the central hospital laboratory of each institution.

Analgosedation depended on the criteria of the clinician in charge of the patient. It was maintained using continuous infusions of different drugs, according to routine clinical practice and the usual department protocol of each institution. These protocols include midazolam (0.03–0.2 mg kg h^1^), propofol (0.5–4 mg kg h^1^), morphine (0.5–5 mg h^−1^), fentanyl (1–10 mcg kg h^−1^), remifentanil (0.05–0.2 mcg kg min^−1^), or dexmedetomidine (0.4–1.4 mcg kg h^−1^). In cases where neuromuscular blocking was needed, it was used a continuous perfusion of cisatracurium (0.06–0.3 mg kg h^−1^) or rocuronium (0.3–0.6  mg kg h^1^). Likewise, mechanical ventilation was personalized for each patient according to the severity of illness and gasometric analytical parameters, as per department protocols.

Subsequently, within 30 days after all these data collections, the patient’s electronic medical record was checked and the date of hospital admission, date of admission to ICU, date of discharge to a hospital facility, survival days, or date of death was recorded. According to the 30 days mortality after data collection, the patients were categorized into the non-survivor group and the survivor group.

### Sample size

Patient inclusion was performed by sequential review of cases in a predetermined 12-month recruitment period from September 2020 to September 2021. In terms of post-hoc power calculations, a sample size of 112 subjects yields more than 95% power to declare a significant difference in the distribution of ANIm and Energy (SDNN) scores assuming the distributed variables estimated in the study.

### Statistical analysis

To analyze the data, continuous variables normality was assessed using a Shapiro–Wilk test. Normally distributed variables are expressed as mean and standard deviation. Non-normally distributed variables are expressed as median and 1st; 3rd quartile. Categorical variables are expressed as frequency. The difference in continuous variables between survival and deceased groups was assessed using a Student T-Test or a non-parametric Mann–Whitney test according to normality. The difference in categorical variables between survival and deceased groups was assessed using a Chi-square (χ^2^) or exact Fisher test. Difference between variables was assessed using a Box Plot and receiver operating characteristic curves (ROC) were assessed for variables showing significant differences. The correlations between variables were assessed with a Pearson test. p-value less than 0.05 was considered statistically significant. To evaluate the ability to discriminate survival from deceased patients, a ROC analysis was performed for any variables showing a p < 0.05. The limit value, as well as sensitivity, specificity, positive predictive, and negative values, were determined for any variable showing an AUC > 0.7. Finally, a multivariate discriminant analysis including ANIm, Energy, age, SOFA, and RASS was performed. Apple Numbers version 10.3.9 was used to collect data and all statistical analyses were performed using the SPSS statistics 20.0 (IBM, Armonk, NY) software.

### Ethics approval and consent to participate

The study was approved by the Ethical and Research Committee of Mostoles General University Hospital (Madrid, Spain), registered No. 2020/035. This study was performed in line with the principles of the Declaration of Helsinki and written informed consent was obtained from all the institutions and all the subjects by the legal designees.

## Results

During the study period, 112 patients were collected in five hospitals from Mexico, Colombia, France, and Spain. Patients were divided into two groups, the survival group (n = 55) and the deceased group (n = 57), depending on the 30 days mortality. Of these 112 patients, 55 were female (49.1%) and 57 were male (50.9%); the mean age was 61 years (range 18–85 years).

The survival and deceased groups showed demographic differences in age, the use of neuromuscular blockers, noradrenaline, anticoagulants, and antibiotics; and type of mechanical ventilation, volume-controlled ventilation, and pressure-controlled ventilation as shown in Table 1.

**Table 1:** Homogeneity and comparison of demographic and characteristics data between groups.

	Survivor group n = 55	No—survivor group n = 57	p-value
Sex (Female)	19 (34.5%)	10 (17.5%)	0.04*
Sex (Male)	36 (65.5%)	47 (82.5%)	
Age (years)	56.75 (± 17.5)	65.46 (± 12.2)	0.003*
Weight (kg)	80.1 (± 15.6)	79.7 (± 14.1)	0.9
Height (m)	1.65 (± 0.09)	1.67 (± 0.08)	0.263
IMC (kg m^−2^)	28.0 (25.8 ; 31.7)	29.0 (24.8 ; 31.5)	0.951
Heart disease	21 (38.2%)	23 (40.3%)	0.814
Beta blockers	1 (1.8%)	4 (7.1%)	0.364
Lungs disease	4 (7.27%)	11 (19.2%)	0.062
Immunodeficiency	2 (3.63%)	7 (12.2%)	0.162
Diabetes	15 (27.3%)	17 (29.8%)	0.765
Hepatic disease	1 (1.8%)	3 (5.26%)	0.618
Renal disease	3 (5.45%)	6 (10.5%)	0.490
Obesity	16 (29.1%)	19 (33.3%)	0.628
Neurologic disease	3 (5.45%)	8 (14%)	0.127
Midazolam	21 (38.2%)	24 (42.1%)	0.672
Propofol	35 (63.6%)	30 (52.6%)	0.238
Dexmedetomidine	11 (20%)	9 (15.7%)	0.561
Ketamine	1 (1.8%)	2 (3.5%)	1.000
Fentanil/Sufentanil	36 (65.5%)	39 (68.4%)	0.739
Remifentanil	6 (10.9%)	4 (7.1%)	0.524
Morfine	7 (12.7%)	13 (22.8%)	0.164
Neuroleptic	3 (5.45%)	3 (5.26%)	1.000
Neuromuscular blocker	19 (34.5%)	34 (59.6%)	< 0.001*
Noradrenaline	22 (40%)	33 (57.8%)	0.004*
Dobutamine	6 (10.9%)	3 (5.26%)	0.492
Corticoids	36 (65.5%)	42 (73.6%)	0.905
Anticoagulants	38 (69.1%)	52 (91.2%)	0.011*
Antibiotic	25 (45.5%)	41 (71.9%)	0.015*
Ventilation mode (VCV)	30 (54.5%)	43 (75.4%)	0.020*
FiO2	0.64 (± 0.20)	0.67 (± 0.24)	0.499
Tidal volume (ml)	425 (± 54.89)	435.2 (± 76.15)	0.449
Respiratory rate (rpm)	23.5 (± 5.33)	24.4 (± 5.31)	0.410
PEEP (cmH_2_O)	8.88 (± 2.553)	9.78 (± 2.476)	0.086
PaO2 (mmHg)	78.5 (69.5 ; 96.5)	82.9 (72.0 ; 98.0)	0.698
PaFi	159.5 (± 80.0)	139.1 (± 62.0)	0.134
Supine position	41 (74.5%)	48 (84.2%)	0.206
SOFA score	7.80 (± 2.870)	9.23 (± 3.365)	0.017*
RASS score	-4.0 (-5.0 ; -3.0)	-4.0 (-5.0 ; -4.0)	0.144
CRP (mg dl^−1^)	28.8 (6.7 ; 130.0)	30.5 (16.3 ; 176.0)	0.263
IL-6 (pg ml^−1^)	358.1 (± 578.6)	678.7(± 545.4)	0.151
Procalcitonin (ng ml^−1^)	0.35 (0.15 ; 0.60)	0.3 (0.2 ; 0.7)	0.313
ANIm	66.39 (± 15.1)	74.1 (± 17.1)	0.013*
Energy (s)	0.587 (± 0.432)	0.322 (± 0.351)	0.001*

Basic descriptives and tests for the demographic and characteristics variables for each group. Absolute (N) and relative (%) frequencies for the qualitative variables. Normally distributed quantitative variables are expressed as Mean (Standard deviation) and non-normally distributed quantitative variables are express as median and quartiles (1st; 3rd). P values were calculated using Student's t-test and Mann–Whitney tests.

SOFA, Sequential Organ Failure Assessment; RASS, Richmond Agitation-Sedation Scale; CRP, C-reactive protein; IL-6, Interleukin-6; VCV, Volume-controlled ventilation; FiO2, Fraction of inspired oxygen; PEEP, positive end expiratory pressure; PaO2, partial pressure of oxygen in arterial blood; ANIm, mean analgesia nociception index.

*Significance defined as p-value < 0.05.

### Heart rate variability

**Figure 2:**
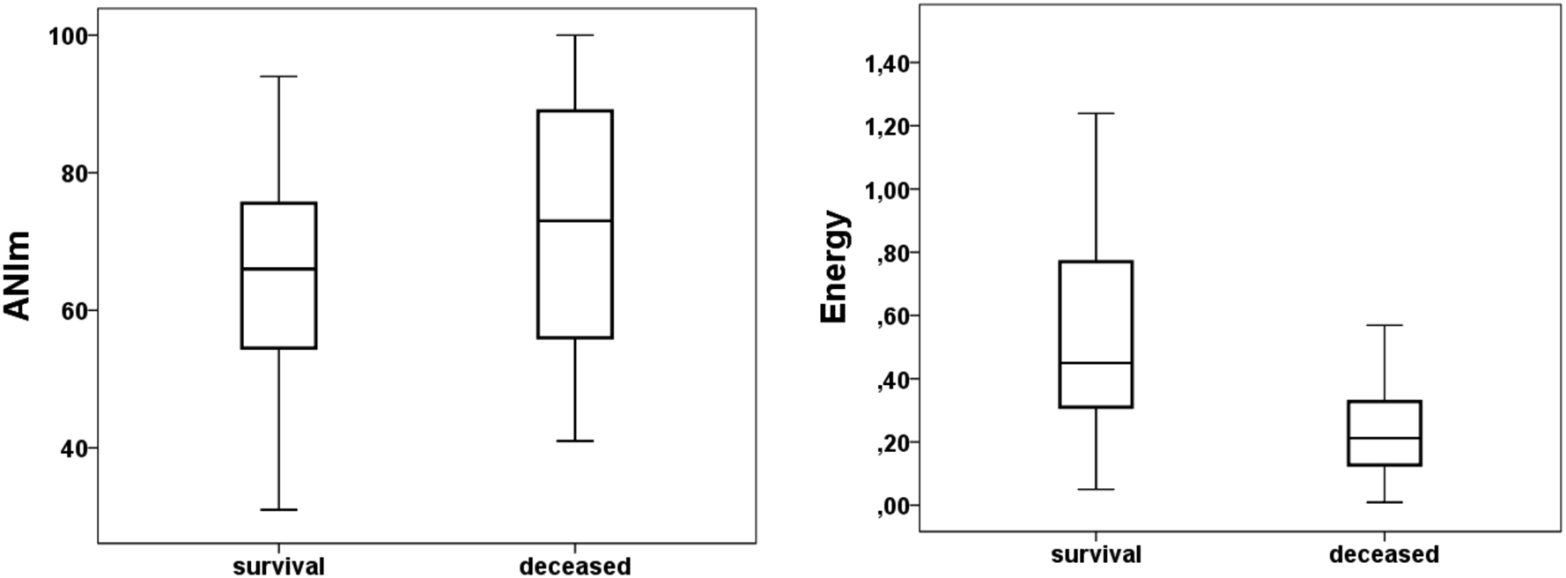
Box Plot for ANIm (left) and Energy (right). Box plot represents the median values of ANIm and Energy in both groups. ANIm, mean analgesia nociception index.

Both ANIm and Energy (SDNN) figures were statistically different between groups and correlated with the disease severity. The ANIm figures were higher in the deceased group, with p = 0.013; survival group 66 (15) and deceased group 74 (17) (Fig. 2). Likewise, Energy (SDNN) was lower in the deceased group, with p = 0.001; survival group 0.587 (0.432) s and deceased group 0.322 (0.351) s (Fig. 2).

No correlation was found between age and ANIm (r = − 0.036, p = 0.710) or between age and Energy (r = − 0.186, p = 0.05).

Regarding the relationship between inflammatory cytokines and HRV values, it was found that Energy (SDNN) levels were not statistically correlated with any of them; CRP (r = − 0.017, p = 0.88), IL-6 (r = − 0.347, p = 0.076), procalcitonin (r = − 0.71, p = 0.565). Higher ANIm levels were statistically correlated with higher CRP (r = 0.349, p = 0.001), but there was no such relationship with the rest of the cytokines, such as IL-6 (r = 0.350, p = 0.073) or procalcitonin (r = 0.082, p = 0.506).

When we analyzed the SOFA score between groups, there were significant differences between them, as expected, with p = 0.017; survival group 7.80 (2.87) and deceased group 9.23 (3.36). In our sample of patients, neither ANIm (r = − 0.099, p = 0.300) nor Energy (SDNN) (r = − 0.072, p = 0.450) were correlated with the SOFA score.

For the relationship between the level of analgosedation, measured by the RASS score, we found that higher ANIm values were correlated with deeper levels of sedation (r = 0.246, p = 0.009). However, we found no relationship between Energy and RASS (r = − 0.099, p = 0.301).

### Predictive analysis for mortality in COVID-19 critically ill patients

If we analyze the relationship between HRV variables and the prognostic values for survival, we found that higher Energy levels, i.e., higher SDNN, were correlated with higher survival days (r = 0.388, p = 0.009). We found no correlation between Energy and days to extubation or days to discharge in the survival group. Otherwise, ANIm was not correlated to any prognostic value (Table 2).

**Table 2:** Correlation between prognostic variables with ANIm and Energy between groups.

	ANIm		Energy	
	Pearson's r	p-values	Pearson's r	p-values
Survival days	− 0.146	0.337	0.388	0.009*
Days to extubation	− 0.049	0.832	0.106	0.649
Days to discharge	− 0.051	0.750	0.144	0.363

Pearson's correlation coefficient and HRV variables for each group; ANIm, mean analgesia nociception index.

*Significance defined as p-value < 0.05.

**Figure 3:**
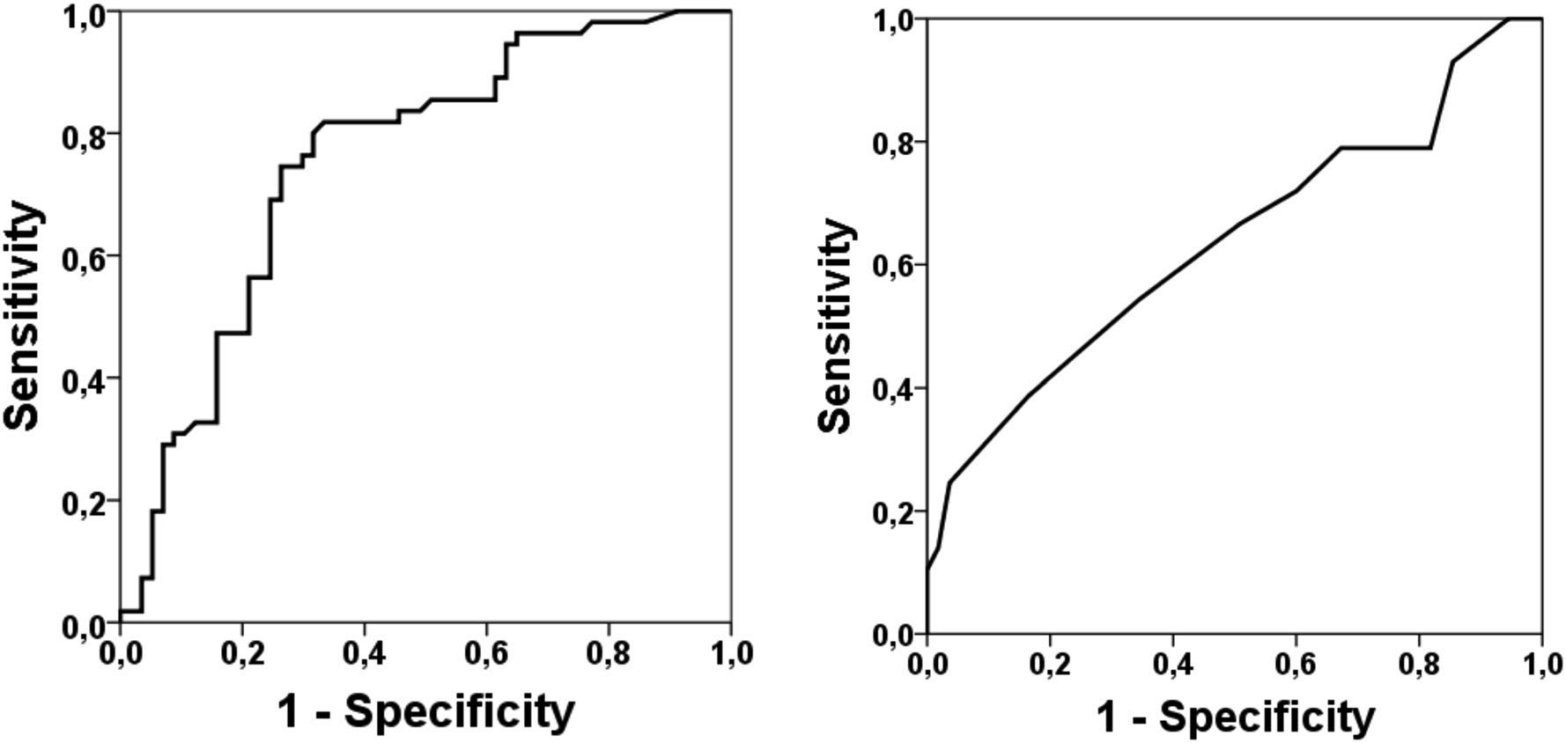
Comparison of ROC curves for Energy (left) and SOFA scale (right). ROC curves demonstrate the ability of Energy (SDNN) to discriminate the mortality with an AUC = 0.755 at an Energy threshold of 0.31 s (sensitivity 71.9%, specificity 74.5%, a positive predictive value 74.5%, predictive negative value 71.9%) compared with the ability of SOFA scale to discriminate the mortality with an AUC = 0.636. AUC, area under the curve.

To attempt to predict the risk of mortality and for the calculation of diagnostic accuracy, the corresponding ROC curves were analyzed for Energy (SDNN), and SOFA scale. For Energy, we found that a limit value of 0.31 s predicted mortalities with a sensitivity of 71.9%, a specificity of 74.5%, a positive predictive value of 74.5%, and a negative predictive value of 71.9% (Fig. 3).

If we compare the ROC curve of Energy (Fig. 3 left) with the AUC = 0.755 and the ROC curve of the SOFA score (Fig. 3 right) with the AUC = 0.636, we discovered that the predictive accuracy of Energy. i.e., SDNN, in predicting mortality is higher than the SOFA score in this kind of patient.

**Figure 4:**
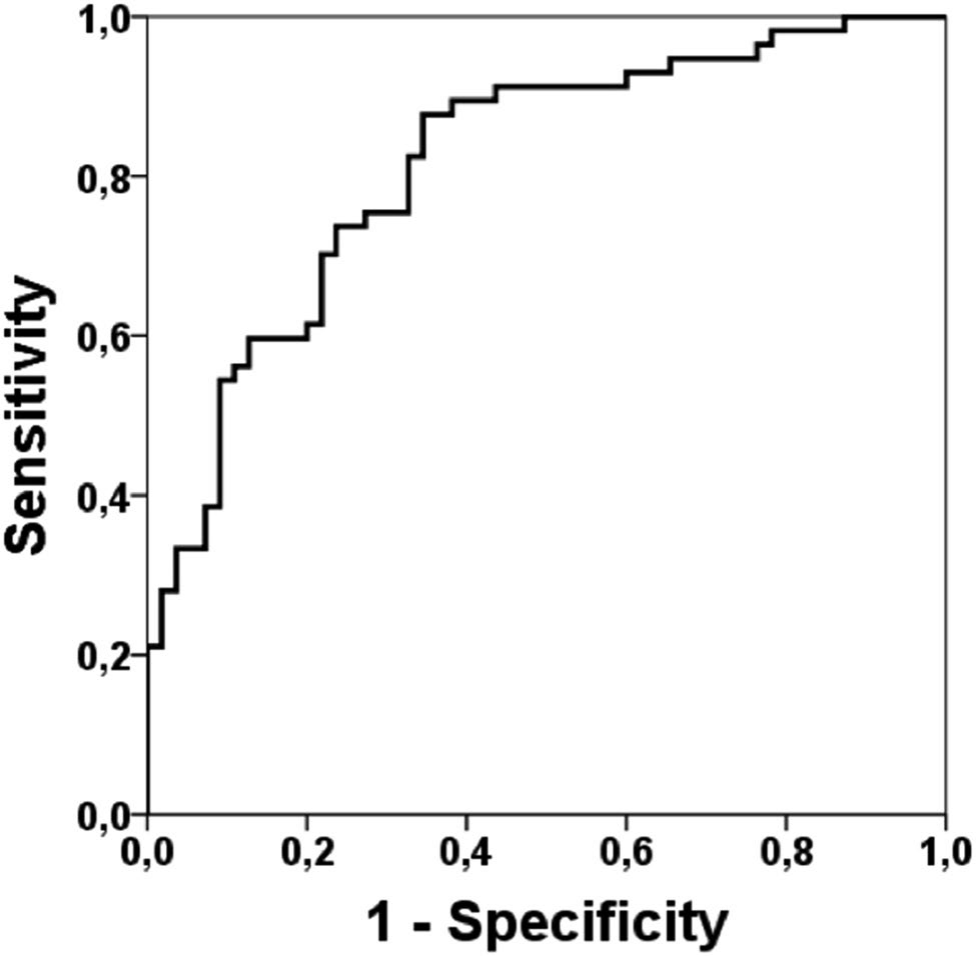
ROC curve for the multivariate model including Age, RASS scale, SOFA scale, Energy and ANIm. ROC curve demonstrates the ability of multivariate model to discriminate the mortality with an AUC = 0.817 (sensitivity 73.7%, specificity 75.5%, a positive predictive value 75%, predictive negative value 73.2%). 74.1% of the records were correctly classified. AUC, area under the curve.

Our multivariate analysis was performed using the ANIm, SDNN (Energy), age, SOFA score, and the RASS scale. In this case, the sensitivity increased to 73.7%, the specificity increased to 75.5%, the positive predictive value increased to 75%, the negative predictive value increased to 73.2% and the AUC was 0.817. For this multivariate analysis, 74.1% of the records were correctly classified (Fig. 4).

## Discussion

To our knowledge, this study is the first prospective, multicenter, international study that studies the monitoring of HRV as a predictor of severity, survival, and mortality in critically ill patients on mechanical ventilation with coronavirus. The results of this research are consistent with and clarify the data found in our previous pilot study^9^.

### Autonomic dysregulation in the patient with SARS-COV-2

Agreeing with our results^9^, Mol et al. and Pan et al.^7,8^ concluded that a decrease in HRV, measured by SDNN, can be used as a biomarker to predict severity at different stages of the COVID-19.

The cause of the autonomic dysfunction and the decrease in HRV seems to be multifactorial^8,20^. As we can see in our results and other studies, SDNN (Energy) is a marker of health status^20^ that also may vary with age^7^, with the neurotrophic effect of SARS-CoV-2 itself^1^, with cardiological autonomic involvement^21^, with bacterial superinfection^22^, and with the evolution of the COVID-19^20,23–25^.

These studies conclude that in the first acute phase of the disease, the most severe patients have a predominance of the sympathetic nervous system compared with the parasympathetic, with a higher ratio of low to high-frequency power (LF/HF)^7,8^. This first acute phase would correspond to an overactive immune response and excessive hyper-inflammation that follows the incubation of SARS-CoV-2^7^, which is mediated by the sympathetic nervous system^26,27^. During this pro-inflammatory response, there is a large discharge of inflammatory cytokines, with activation of the hypothalamic–pituitary–adrenal axis and a large adrenergic and cortisol release^2,3,28^.

After this first proinflammatory stage, and during the post-acute compensatory anti-inflammatory response (CARS)^26–28^, there is a substantial adrenergic and corticosteroid hormone deficiency, with sympathetic nervous system depletion and immune anergy, which results in the immune system being ineffective in controlling the virus, leading to fatal multi-organ failure^1–3,25^.

This can be confirmed in our results, where in the last phase of the disease there is a depletion of the ANS with lower Energy (SDNN). On the other hand, the higher ANIm observed in the deceased group compared with the survivors should be explained by a depletion of the sympathetic nervous system leading to a predominance of the parasympathetic system.

This pattern can also be seen in patients with poor prognosis and higher mortality with severe sepsis^29^, postoperative patients with acute respiratory distress syndrome (ARDS)^30^, patients after myocardial infarction^31^, chronic heart failure^32^, left ventricular dysfunction^33^, polytraumatized patients^34^, or in chronic diseases^35^.

### Comparative analysis of energy and SOFA scale as a predictor of mortality

The SOFA score is an international scale, widely used, and currently considered the gold standard for predicting multiorgan failure in critically ill patients with sepsis^19^. It is a multiparametric scale, which requires laboratory analytical parameters which are often not available at the patient's bedside^18,19^.

Our findings suggest that the Energy (SDNN) can predict mortality with more specificity and sensitivity in this type of patient when it is compared with the SOFA scale. In addition, the Energy values were directly correlated with survival days, i.e., the lower the Energy, the lower the survival days.

Therefore, the analysis of HRV, and more specifically the Energy value, seems to be a non-invasive and more effective way of assessing survival than the SOFA scale, which can be useful as a triage tool or as a complement to inform therapeutic decisions.

### ANIm, RASS scale, and analgosedation

The RASS scale, although subjective and physician-dependent, is the scale, along with hemodynamic parameters, most widely used in critical care units to assess the degree of analgosedation and agitation in critically ill patients^18,19^. However, pain scales used in critically ill patients, such as the Campbell scale, require a certain degree of consciousness and muscle tone, and they are, therefore, not recommended in patients under deep sedation and neuromuscular blockade^36^.

In our study, the ANIm value correlated directly with the RASS scale, i.e., those patients with greater sedation according to the RASS scale had higher ANIm values. The use of HRV is already widely used in the operating room to monitor the imbalance between nociception and anti-nociception, and numerous articles, such as the study by Boselli et al. in patients with COVID-19^37^, conclude that the use of spectral analysis of HRV with ANIm using the ANI monitor can be a reliable biomarker to monitor nociception in the critically ill patient with COVID-19.

### ANS control and analgosedation

Moreover, as suggested by Upton et al.^38^ in their clinical trial and by most international guidelines^6,36,39^, excessive analgosedation in both the critically ill and the surgical patient can lead to significant depletion of the sympathetic nervous system, and ultimately to greater complications and worse disease outcome.

As previously mentioned, the modulation and control of the ANS are multifactorial. There are different mechanisms to modulate the autonomic nervous system of our patients such as vagus nerve stimulation^40^, transcranial stimulation^41^, control of inspiration and expiration^42^, or even by the control of the nociceptive autonomic medullary circuit^13,26–28,40^.

It is possible to modulate by analgosedation drugs the nociceptive autonomic medullary circuit, responsible for activating the nucleus of the solitary tract. This nucleus of the solitary tract, in the case of any nociceptive, inflammatory, or infectious stimulus, is the subcortical center that controls the sympathetic response, and therefore, the pro-inflammatory response. It also controls the activation of the vagus nerve and the consequent activation of the anti-inflammatory cholinergic chain^13,26,28^.

In our study, we have seen that a significant depletion of the sympathetic nervous system and an excess of parasympathetic activity predicts a worse progression of the disease and higher mortality. Both overdosing and underdosing of analgosedation can, therefore, lead to worse patient outcomes.

We must individualize analgosedation for each patient taking into account several objective factors. Firstly, the state of the CNS, using EEG spectral analysis, minimizes the possible neurological damage derived from an excess of cortical suppression^36,39,42^. Secondly, we must monitor the autonomic nervous system using HRV to determine the state of the sympathetic and parasympathetic nervous system, i.e., ANIm, and maintain a balance and homeostasis between both. And thirdly, to monitor the severity and fragility of the patient, measured through the Energy (SDNN), to titrate and adjust the dosage more precisely in those patients who were more fragile and with greater depletion of the ANS^7–9,38^.

As suggested by different studies^43,44^ and international protocols^36,39^, we should promote multimodal analgosedation in critically ill patients, minimizing the use of lipophilic opioid drugs, like fentanyl, and using adjuvant drugs, such as ketamine or dexmedetomidine, to control this autonomic response.

It is well known that ketamine has a certain sympathomimetic effect and could, therefore, be useful as an adjuvant in patients with hemodynamic instability and certain autonomic depletion^44^. On the other hand, dexmedetomidine, an alpha-2-agonist drug that acts on the locus ceruleus inhibiting the "fight or flight" adrenergic response, is an important sympatholytic, which should be reserved in critical patients in an early stage with a significant predominance of the sympathetic nervous system, or a later stage in the weaning phase^39,43^, limiting it in more fragile patients with worse prognosis, with less Energy (SDNN).

### Limitations

Despite its strengths, there may be some possible limitations in this study that must be taken into account. Primarily, this is an observational study, with no randomization possibility. Furthermore, our study is a multicenter study and therefore the different analgosedation strategies depend on the clinical criteria and the protocols of each hospital center. Mechanical ventilation was personalized for each patient according to the severity of illness which can constitute a bias in ANI interpretation. However, Jeanne et al.^16^ demonstrated that the normalization process strongly limits the effect of the respiratory volume and that the graphical assessment (i.e. Area under the envelopes computation) strongly limited the effect of the respiratory rate.

In future studies, we plan to analyze in a randomized manner whether or not monitoring HRV improves the titration of analgosedation and improves the evolution of critically ill patients. Furthermore, a retrospective analysis including other HRV common markers (HF, LF, HFnu) would allow us to better explain the physiopathological link between HRV and COVID-19 patients' severity. The inclusion and calculation of other HRV metrics in a future analysis should lead to better sensitivity/specificity in predicting mortality and further validate the present results.

## Conclusion

According to the results of our study, we conclude that HRV monitoring in critically ill patients with coronavirus allows predicting, with high sensitivity and specificity, survival days and mortality through Energy. We demonstrated that the use of the Energy (SDNN) value by itself, and together with other parameters by multivariate analysis with age, the RASS scale, and the ANIm value, allows predicting with higher specificity and higher sensitivity the mortality of critically ill patients with COVID-19 in comparison with the SOFA scale.

We further concluded that frequency domain parameters such as LF (sympathetic) and HF (parasympathetic) also vary according to the stage and severity of the disease. According to our sample, those patients with greater severity (i.e., those patients who did not survive) showed a predominance of relative parasympathetic activity (i.e., ANIm value).

In summary, on the basis of our results and those of other studies, the recommendation to control the immune and inflammatory system by modulating the ANS of our critically ill patients needs to be considered. To this end, we suggest reviewing our analgosedation protocols in critically ill patients, controlling the balance between sympathetic and parasympathetic by analysis of HRV. We also encourage individualizing the dosage of analgosedation for each patient, titrating and using the minimum effective dose, avoiding under- and overdosing.

## Data Availability

The datasets used and analyzed during the current study are available from the corresponding author on reasonable request.

## Abbreviations

COVID-19: Coronavirus Disease 2019
HRV: Heart Rate Variability
ANS: Autonomic Nervous System
CRP: C-reactive protein
SOFA: Sequential Organ Failure Assessment score
RASS: Richmond Agitation-Sedation scale (RASS)
PCR: Polymerase Chain Reaction
ANI: Analgesia Nociception Index
SDNN: Standard Deviation of all Normal R–R Intervals
RRi: R–R Intervals
RRmean: R–R Intervals Mean
HF: High-frequency
LF: Low-frequency
VLF: Very low-frequency
RSA: Respiratory Sinus Arrhythmia
AUC: Area Under the Curve
ROC: Receiver Operating Characteristic Curves
ARDS: Acute Respiratory Distress Syndrome
BIS: Bispectral index
CNS: Central Nervous System
